# Different Transcriptional Responses from Slow and Fast Growth Rate Strains of *Listeria monocytogenes* Adapted to Low Temperature

**DOI:** 10.3389/fmicb.2016.00229

**Published:** 2016-03-01

**Authors:** Ninoska Cordero, Felipe Maza, Helen Navea-Perez, Andrés Aravena, Bárbara Marquez-Fontt, Paola Navarrete, Guillermo Figueroa, Mauricio González, Mauricio Latorre, Angélica Reyes-Jara

**Affiliations:** ^1^Laboratorio de Microbiología y Probióticos, Instituto de Nutrición y Tecnología de los Alimentos, Universidad de ChileSantiago, Chile; ^2^Department of Molecular Biology and Genetics, Istanbul UniversityIstanbul, Turkey; ^3^Laboratorio de Bioinformática y Expresión Génica, Instituto de Nutrición y Tecnología de los Alimentos, Universidad de ChileSantiago, Chile; ^4^Center for Genome Regulation (Fondap 15090007), Universidad de ChileSantiago, Chile; ^5^Mathomics, Center for Mathematical Modeling, Universidad de ChileSantiago, Chile

**Keywords:** *Listeria monocytogenes*, low temperature, growth rate, global gene expression, motility

## Abstract

*Listeria monocytogenes* has become one of the principal foodborne pathogens worldwide. The capacity of this bacterium to grow at low temperatures has opened an interesting field of study in terms of the identification and classification of new strains of *L. monocytogenes* with different growth capacities at low temperatures. We determined the growth rate at 8°C of 110 strains of *L. monocytogenes* isolated from different food matrices. We identified a group of slow and fast strains according to their growth rate at 8°C and performed a global transcriptomic assay in strains previously adapted to low temperature. We then identified shared and specific transcriptional mechanisms, metabolic and cellular processes of both groups; bacterial motility was the principal process capable of differentiating the adaptation capacity of *L. monocytogenes* strains with different ranges of tolerance to low temperatures. Strains belonging to the fast group were less motile, which may allow these strains to achieve a greater rate of proliferation at low temperature.

## Introduction

Foodborne pathogens are a worldwide concern for human disease and public health. One of these pathogenic microorganisms is *Listeria monocytogenes*, which can resist various important barriers applied in food chain production, such as refrigeration temperatures. In humans *L. monocytogenes* causes listeriosis, the third cause of death after non-typhoidal *Salmonella* sp. and *Toxoplasma gondii* infections (Scallan et al., 2011), producing manifestations that range from febrile gastroenteritis to more severe, invasive disease (Franciosa et al., 2005; Hernandez-Milian and Payeras-Cifre, 2014).

Several studies have shown that the versatility of *L. monocytogenes* to proliferate in different food matrices lies in its ability to grow in a broad spectrum of temperatures, between 1 and 45°C (Walker et al., 1990; Azizoglu et al., 2009). After exposure to low temperatures the bacterium passes through two stages, acclimation (characterized by cell arrest) and adaptation (where cells are able to grow, but at a slower rate; Barria et al., 2013). Previous reports have evaluated the mechanisms of *L. monocytogenes* involved in acclimation and growth at low temperature (Tasara and Stephan, 2006; Chan et al., 2007; Chan and Wiedmann, 2009; Soni et al., 2011). The low temperature stress decreases the metabolic rate and response of the bacterium, changing the gene expression pattern and improving cell fitness at a low temperature. This change produces several modifications, including membrane composition (Mastronicolis et al., 2006; Yoon et al., 2015), synthesis of cold shock proteins (Thieringer et al., 1998; Schmid et al., 2009; Chaikam and Karlson, 2010) and synthesis of transporters that facilitate uptake of osmolytes that are cryoprotective nutrients (Angelidis and Smith, 2003).

All these capacities indicate the existence of different mechanisms involved in the acclimation and adaptation phases that are able to respond against different temperatures (Bresolin et al., 2008), suggesting the presence in *L. monocytogenes* strains of plasticity genome events, plasmid elements or transcriptional regulation such as sigma factor protein (Becker et al., 2000), CodY, proteins encoded by lmo0287 (Chan et al., 2007) and lmo0501 (Michel et al., 2011). These sensors convert the input signals into gene regulation, allowing the bacterium to survive and adapt its metabolism to a new environment and maintain its homeostasis (Chan et al., 2007; Metselaar et al., 2015).

Microarray expression data show that *L. monocytogenes* under low temperature activates mechanisms involved in cold protection, and induces the expression of genes related to translation, transcription, cell division, basal metabolism, and energy production (Garmyn et al., 2012; Rantsiou et al., 2012; Durack et al., 2013). These findings reveal that the adaptation to low temperature can be classified as a complex response (Latorre et al., 2014), where the bacterium must adjust its transcriptional response at the system level in order to maintain cellular homeostasis.

Using global transcriptomic assays, we explored whether strains of *L. monocytogenes* with different growth rates at a low temperature (8°C) present particular or common transcriptional patterns activated during the adaptation to low temperature.

## Materials and Methods

### Bacterial Strains and Culture Media

**FIGURE 1:**
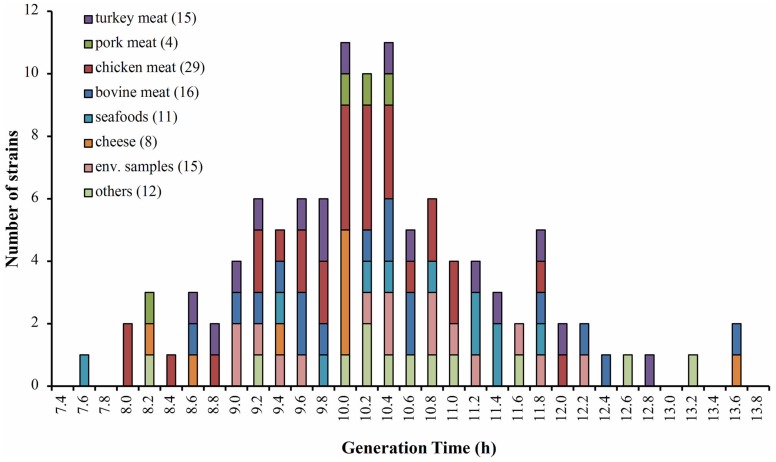
**Distribution of generation times (Gt) for 110 isolates of *Listeria monocytogenes* growing at 8°C**. The colors indicate the food matrices from where the strains of *L. monocytogenes* were isolated. In parentheses the number of isolates by matrix.

This study used 110 isolates of *L. monocytogenes*, whose origins are described in **Figure 1** and **Supplementary Table S1**. We confirmed that the isolates were effectively *L. monocytogenes* by polymerase chain reaction (PCR) using primers specific to the *iap* gene described by Bubert et al. (1992). Isolates were stored in skimmed milk (20%) at -80°C until required. To recover the strains we used Oxford selective agar (Oxoid, Basingstoke, UK). Growth curves were performed in Tripticase Soy Broth (BBL, Becton Dickinson, USA) containing 0.6% yeast extract (Oxoid, Basingstoke, UK; TSBYE). All cultures were carried out at 30 or 8°C, according to the test indications.

### Growth Curves

We harvested a single colony of *L. monocytogenes* from Oxford agar with a sterile loop and cultured it in 5 mL of TSBYE at 30°C, 160 rpm overnight (Gehring et al., 2014). The following day 100 μL of the culture was transferred to fresh TSBYE medium (5 mL) and incubated at 8°C for 4 days to adapt the isolates to low temperature. Then, 30 mL of the medium was adjusted to an optical density of 0.05 at 600 nm (OD_600__nm_) and grown at 8°C at 160 rpm. Growth curves at 30°C were performed from an overnight culture in TSBYE at 30°C which was transferred to the same fresh medium at a starting OD_600__nm_ of 0.05 and grown at 30°C with 160 rpm. Temperatures of 8 and 30°C were selected based on previous studies (Faleiro et al., 2003; Mastronicolis et al., 2006). Growth curves were monitored by reading OD_600__nm_. The OD conversion to Log CFU was generated using the fitted curve and the generation time calculated for each strain at 8 and 30°C (Madigan et al., 2008).

### Genotype Characterization

We selected the six slowest and six fastest growing strains at 8°C to be genetically characterized by pulse field gel electrophoresis (PFGE). PFGE was carried out following the CDC standardized PulseNet protocol for *L. monocytogenes* (http://www.cdc.gov/pulsenet/protocols/pulsenet_listeria_protocol%20.pdf), using the restriction endonuclease *Asc*I (New England Biolabs, Ipswich, MA, USA). We analyzed the resulting PFGE pattern using the Gel ComparII Software (Bionumerics 2011 Applied Maths NV) according to Foerster et al. (2012). To normalize the images we loaded *Xba*I-digested standard strain *Salmonella* Enterica serovar Braenderup H9812 in two lanes in each gel. We built a UPGMA dendrogram using a Dice coefficient with 1.0% tolerance (Foerster et al., 2012).

### RNA Extraction

For microarray experiments at low temperature, fast and slow strains were previously adapted for 4 days at 8°C, 160 rpm, then the cultures were diluted in fresh TSBYE medium adjusting to OD_600__nm_ = 0.05. Then fast and slow strains were cultured for different times to obtain cells that were at the early logarithmic phase (OD_600__nm_ = 0.3). For cultures at 30°C, bacteria were grown overnight and the next day transferred to fresh TSBYE medium and adjusted to OD_600__nm_ = 0.05. Bacteria were cultured at 30°C until they reached OD_600__nm_ = 0.3. To evaluate the effect of low temperature on the expression levels for flagellar genes by qPCR, bacteria (fast and slow strains) growing at early exponential phase at 30°C (OD_600__nm_ = 0.3) were transferred to fresh culture medium at 8°C, 160 rpm. Bacterial cells were collected after 1, 2, 4, and 24 h. For bacteria adapted to low temperature, the same growth conditions used for the microarray assay were used.

Total RNA was extracted from the selected strains using an RNeasy kit (Qiagen); its quantity and quality were checked with a NanoDrop ND-1000 and Agilent 21000 BioAnalyzer. This RNA sample was used for microarray and qPCR experiments.

### Global Expression Analysis

Two *L. monocytogenes* strains belonging to the fast group (LIST 2-2 and APA 13-2) and two strains of the slow group (AL 157-12 and BS 3-2) were selected for global gene expression analysis (see details in **Supplementary Table S1**). We determined global expression using microarray slides manufactured by Microarrays, Inc. Each slide contained 70-mer probes representing 2,857 ORF of *L. monocytogenes* EDG-e in triplicate. We extracted RNA from two biological and two technical replicas, and synthesized cDNA labeled with Cy3 and Cy5 (GE Health Care). The temperature of 8°C was considered the experimental temperature and 30°C was the control temperature. The samples were hybridized on the slides according to the recommendations of Microarrays, Inc. The slides were scanned on an Agilent technologies scanner and quantified using the program Scan Array Express (Perkin Elmer).

To determine significant differential expression we used the *limma* library from the statistical package R. We evaluated the quality of each spot using the Qcom index defined in Wang et al. (2001) and discarded the spots where this index was less than 0.6. We used the normal-exponential background correction method and print-tip-loess color bias correction on each slide. Finally, we normalized all slides using the A-quantile method. Following the *limma* protocol, we determined the significance of each spot’s differential expression using a Student’s *t*-test and an empirical Bayes estimation. We considered that a gene was differentially expressed when the absolute log-fold change was greater than 1 (*p*-value < 0.05).

We determined which COG categories were overrepresented among the differentially expressed genes using the Fisher exact test and a threshold *p*-value of 0.05. We obtained the metabolic super-pathway for *L. monocytogenes* EDG-g from the MetaCyc database with Taxonomy ID 1639 (Karp et al., 2002). We integrated the microarray data into this metabolic network using the program IPath 2.0 (Yamada et al., 2011). Microarray data are available in **Supplementary Data Sheet S1** and **Supplementary Table S2**.

### Quantitative PCR (qPCR)

Two micrograms of total RNA were reverse transcribed with Moloney Murine Leukemia Virus Reverse (Promega, USA) using random primers (Invitrogen). PCR primers were designed with Primer3 Plus software using the *L. monocytogenes* EGD-e genome sequence (**Supplementary Table S3**). Quantitative PCR and data analysis were performed using the real-time PCR system, LightCycler^®^96 (Roche). Amplification efficiencies were calculated using LinRegPCR software. The relative expression level of each gene was calculated using the 2^–ΔΔCT^ method (Livak and Schmittgen, 2001), using 16S rRNA gene (lmor04) as a reference (Tasara and Stephan, 2007). The results were expressed as the fold change (log2) between 8 and 30°C cultures.

### Motility Assays

Swimming motility for each isolate was individually assessed on TSBYE supplemented with 0.20, 0.25, and 0.30% agar. Briefly, plates were inoculated using a sterilized wooden stick and incubated at 8°C for 10 days. At different times the diameter of the colony was measured and recorded.

## Results and Discussion

### Growth at Low Temperature

The growth at 8°C and 30°C of 110 *L. monocytogenes* isolated from different food matrices was monitored (**Figure 1**). The generation time was calculated for each strain at both temperatures. The results at 8°C showed a wide range of generation times among different strains (5–14 h), which was independent of the origin of the strains. According to the growth rate at 8°C, we classified the lowest 5% and highest 5% of generation times as the fast and slow groups of the strains, respectively (*n* = 6 for each group). The classification of these strains according to their generation time at 30°C was also performed (**Supplementary Table S1**). The strains were not classified in the same group that was observed at 8°C, indicating different behavior regarding adaptation to different temperatures (**Supplementary Figure S1**). These results reveal high variability in the adaptation to low temperature among studied strains, supporting previous data obtained in *L. monocytogenes* isolated from foods (Nufer et al., 2007; Durack et al., 2013).

Fast and slow isolates were characterized to determine their genetic relatedness using PFGE. For this study, isolates that showed PFGE patterns with >90% similarity were defined as the same pulsotype. The results showed 11 pulsotypes in the 12 *L. monocytogenes* strains, revealing high genetic diversity among slow and fast growing strains (**Supplementary Figure S2**). Only two slow growing isolates, AL113 and BS 3-2, both isolated from bovine meat, belonged to the same pulsotype (>90% similarity). These results are in accordance with a previous study that showed high genetic diversity between *L. monocytogenes* strains isolated from foods. Similar to our results, *L*. *monocytogenes* isolates with the same pulsotype have been recovered from the same food matrix (Montero et al., 2015). Finally, the two slowest and two fastest growing strains, with pulsotypes having <80% similarity, were selected to elucidate differences in the global transcriptional response between fast and slow strains of *L. monocytogenes* adapted to low temperature.

### Global Transcriptional Changes in Response to Cold Adaptation

We evaluated putative differences in the transcriptional response between *L. monocytogenes* slow and fast groups both previously adapted to low temperature (4 days). We performed a microarray analysis to compare the transcriptome profiles of each group growing exponentially in TSBYE medium either at 8°C (cold adaptation) or at 30°C (control).

**FIGURE 2:**
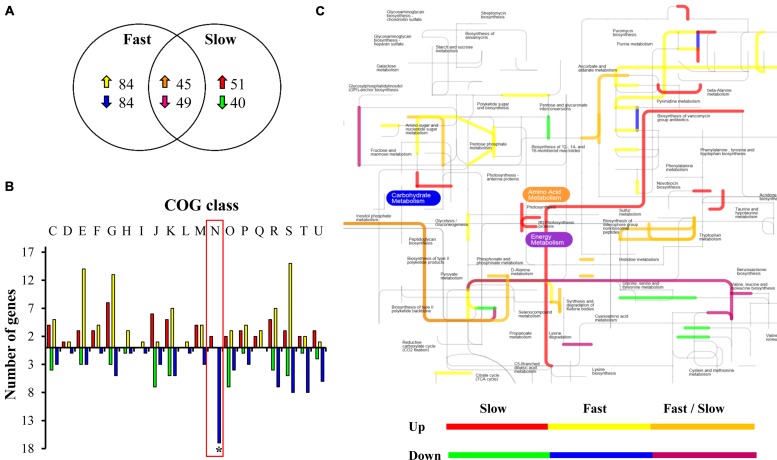
**Analysis of the transcriptional response of *L. monocytogenes* at low temperature**. **(A)** Venn diagram of common and unique sets of genes differentially expressed in *L. monocytogenes* strains belonging to fast or slow groups at 8 vs. 30°C. Up arrows indicate over expressed genes and down arrows, repressed genes. **(B)** Differentially expressed genes in fast and slow groups in response to low temperature grouped by COGs. The genes were classified into 20 COGs based on their predicted function. C: Energy production and conversion; D: Cell cycle control, cell division, chromosome partitioning; E: Amino acid transport and metabolism; F: Nucleotide transport and metabolism; G: Carbohydrate transport and metabolism; H: Coenzyme transport and metabolism; I: Lipid transport and metabolism; J: Translation, ribosomal structure and biogenesis; K: Transcription; L: Replication, recombination and repair; M: Cell wall/membrane/envelope biogenesis; N: Cell motility; O: Post-translational modification, protein turnover, chaperones; P: Inorganic ion transport and metabolism; Q: Secondary metabolite biosynthesis, transport and catabolism; R: General function prediction only; S: Function unknown; T: Signal transduction mechanisms; U: Intracellular trafficking, secretion, and vesicular transport; V: Defense mechanisms. The asterisk indicates a significant difference between the fast and the slow group. **(C)** Metabolic pathway activated in response to low temperature unique and shared between strains belonging to low and high temperature. Fast strains: LIST 2-2 and APA 13-2 and slow strains: AL 157-12 and BS 3-2.

The results revealed that growing at 8°C, compared to growing at 30°C, a total of 262 genes were differentially expressed (up or down regulated) in the fast group and 185 in the slow group, representing 9 and 6.5% of the total genome of *L. monocytogenes*, respectively (**Figure 2A**). A similar number of genes changed their expression in *L. monocytogenes* strain 10403S in response to low temperature (4°C) compared to 37°C (Chan et al., 2007). Furthermore, in *Pseudomonas putida* KT2440, a psycrotolerant microorganism, the comparison of transcriptome profiles of bacteria growing exponentially at 10 vs. 30°C revealed that the low temperature modified the expression of at least 266 genes (about 5% of the genome; Fonseca et al., 2011). In addition, our microarray results revealed that 45 up-regulated genes and 49 down-regulated genes were shared between the groups. This result suggests equivalent changes in gene expression at a low temperature between different strains (conserved core) which give *L. monocytogenes* the ability to grow at refrigeration temperature. We classified the genes that change their expression according to Clusters of Orthologous Groups (COGs). The main shared changes were related to amino acid transport and metabolism (E), carbohydrate transport and metabolism (G) and translation, ribosomal structure and biogenesis (J; **Supplementary Figure S3**).

In previous work, Arguedas-Villa et al. (2014) evaluated the expression level of 9 genes involved in the response to low temperature (lmo0501, *cspA*, *cspD*, *gbuA*, lmo0688, *pgpH*, *sigB*, *sigH*, and *sigL*) in isolates with differential capacity for cold adaptation (Arguedas-Villa et al., 2014). While our results did not show significant differences for all these genes between the fast and slow growth rate strains, our global view allows us to propose other elements that could be involved in differences in cold adaptation in *L. monocytogenes* strains.

The genes observed to be specific and differentially expressed for each group were also analyzed according to their COG categories. We were able to identify different categories responding commonly and particularly between the two groups of strains (**Figure 2B**). In terms of the activated elements at 8 vs. 30°C, in the fast group changes included differential over-expression of a larger number of genes that synthesize proteins involved in COG functional category E (amino acid transport and metabolism), G (carbohydrate transport and metabolism), and S (function unknown). The number of up-regulated genes in the slow group that belong to COG category J (proteins involved in translation, ribosomal structure, and biogenesis) was much greater in relation to the fast group. We identified a large number of genes belonging to COG category N (motility) repressed in the fast group at 8°C. No genes belonging to this category were repressed in the slow group, on the contrary, genes belong to this COG were over expressed in the slow group at low temperature.

Next, we integrated the microarray data into a metabolic network. This strategy allowed us to identify putative pathways activated during the adaptation to low temperature within each group (**Figure 2C**). Slow and fast *L. monocytogenes* strains apparently are inducing routes involved in peptidoglycan synthesis, suggesting an adaptive transposition induced during cold adaptation. This result is in concordance with previous reports in *Escherichia coli*, when cultures of this bacterium exposed to low temperature underwent several changes in cell morphology, largely a shift to septal peptidoglycan synthesis (Pierce et al., 2011).

Both groups of *L. monocytogenes* are repressing metabolic pathways related to the synthesis of the polar (glycine, serine, threonine) and pyruvate families (valine, leucine) of amino acids. These amino acids are important precursors for the synthesis of iso-branched-chain fatty acids, which decrease membrane fluidity (Mansilla et al., 2004). The energy metabolism process was highly induced in the slow group of strains. The energy requirement can be used in different metabolic processes, including *inter alia* the flagellar functioning (Kamp and Higgins, 2009), supporting the idea that motility is a process differentially expressed within both groups of strains (Durack et al., 2013). It is important to state that genome plasticity plays an important role in adaptation to low temperatures (Bresolin et al., 2008). Besides these processes differentially expressed between the slow and fast groups of *L. monocytogenes*, the presence of unique genes, *cis*- and *trans*-mutations, plasmids, transcriptional regulators involved in motility and low temperature (like functional flagellar cluster or mechanism for rapid colonization) may also be implicated in the phenotypes observed (Bresolin et al., 2008; Lauro et al., 2014). Thus the complete genome sequencing of all the strains described in this work will produce important information related to this topic.

### Motility at Low Temperature

Previous studies reported that *L. monocytogenes* regulates flagellar motility according to temperature (Raengpradub et al., 2008). In order to determine whether groups with differential capabilities of adaptation to low temperature are the result of a different transcriptional response, we selected the genes related to motility to evaluate transcriptional response in different stages of acclimation and adaptation to low temperature (8°C).

**FIGURE 3:**
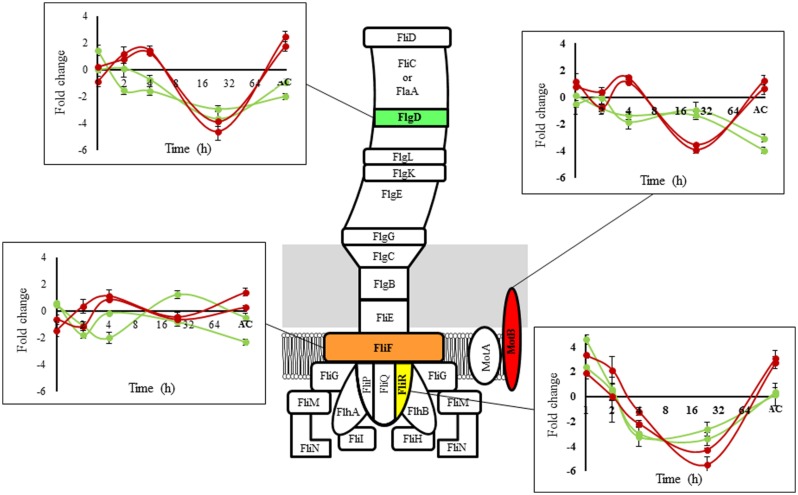
**Schematic flagellar structure of *L. monocytogenes***. Proteins involved are indicated. The graph shows the temporal transcriptional response at 8°C compared to 30°C for selected components (green lines: fast group, red lines: slow group; representing the same strains used in the microarray experiments). AC: Adapted culture (fast group: 4 days (cold adaptation) + 25 h (OD_600__nm_ = 0.3) = total time 121 h; slow group: 4 days (cold adaptation) + 32 h (OD_600__nm_ = 0.3) = total time 128 h).

The selected genes represent different parts of the structure of the flagellum and were encoded in three different operons according to the Prokaryotic Operon DataBase (ProOpDB; Taboada et al., 2012; **Figure 3**). *fliR* and *motB* (operon 394) are genes involved in motor control, *flgD* (operon 397) is involved in flagellum biosynthesis (Casey et al., 2014; Hingston et al., 2015) and *fliF* (operon 398) is involved in flagellum assembly (Bigot et al., 2005). The results revealed that the temporal expression pattern of flagellar genes was similar between strains belonging to the same group at 8°C and presented a variable behavior over time (**Figure 3**). During the acclimation stage (≤4 h) and early adaptation (24 h exposed) to low temperature, differences in the expression of the selected genes between fast and slow strains were not identified. After the adaptation phase (*L. monocytogenes* adapted to low temperature), the genes that encoded for the flagellum structure showed different gene expression in response to low temperature between fast and slow strains. The genes that encoded for MotB and FlgD showed the main differences. These results are in agreement with our microarray analysis, which allowed us to validate the experiment.

**FIGURE 4:**
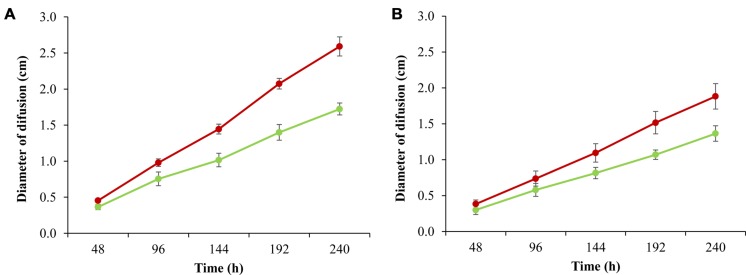
**Motility assay for six fast strains (green lines) and six slow strains (red lines) growing at 8°C in TSAYE medium with 0.20% **(A)**, 0.25% **(B)** agar**. The bars represent one standard deviation. Each point represents the average motility for the strains belonging to each group. Details of the strains in **Supplementary Table S1**.

In addition to confirming this observation, we performed a bacterial mobility assay in all fast and slow strains (**Figure 4**). The results showed that the group of slow strains was significantly more mobile over time at 8°C than the strains belonging to the fast group. Another stress condition, defined as exposure to salt, reduced *L. monocytogenes* motility (Durack et al., 2013), however, to date it has not been described that the capacity to swim may be related to the growth rate.

The reduction in the expression of flagellar genes in *L. monocytogenes* is a phenomenon that has been previously described as an energy saving mechanism to be used in critical metabolic processes (Shen and Higgins, 2006; Hingston et al., 2015). This strategy is apparently used by the fast strains to achieve a greater proliferation rate at low temperature. Recently, Cabrita et al. (2015) described that in the persistent strains isolated from the dairy industry, the *flaA* gene significantly reduces their expression in comparison to sporadic isolated strains when bacteria are growing in late exponential phase at 11°C. The expression of mobility genes is also related to the adhesion capacity of the strains. Mutants for some flagellar genes (*motA*, *flgL*) showed difficulty in attaching to a surface; however, these bacteria were able to form biofilm. These mutants increase their biomass to form biofilm readily and to persist in the environment (Todhanakasem and Young, 2008). In consequence, the results obtained in the present study showed that the machinery involved in flagellar formation in *L. monocytogenes* is transcriptionally repressed in the fast group of strains, as similarly observed in persistent strains.

## Conclusion

Investigations support the hypothesis that *L. monocytogenes* has transcriptional machinery that allows it to grow at a low temperature like that used in the food industry. Previous studies reported that *L. monocytogenes* isolated from different sources display variations in cold adaptability, showing a range of tolerance to low temperatures (Chan and Wiedmann, 2009; Arguedas-Villa et al., 2010, 2014; Durack et al., 2013). Data indicate that genetic diversity or changes in the transcriptional response to cold exposure of the bacteria could explain this observation. In this context, our experimental approach allowed us to identify a particular element among strains with different growth rates at low temperature. Genes related to motility appeared less expressed in fast growing bacteria; similar to the response observed in persistent *L. monocytogenes* strains isolated from the food industry environment. Future studies should evaluate the association between virulence, motility, and growth rate at low temperature for *L. monocytogenes* strains.

## Author Contributions

NC: microarray experiments and data analysis; FM: microarray experiments, primer designs, qPCR, PFGE; HN-P: RNA extraction, qPCR; AA: microarray analysis; BM-F: growth curves; PN: write paper; GF, MG: data analysis; ML and AR-J: Designed the research, analysis of results and write the paper.

## Conflict of Interest Statement

The authors declare that the research was conducted in the absence of any commercial or financial relationships that could be construed as a potential conflict of interest.

## Supplementary Material

FIGURE S1 **Generation time (Gt) distribution at 8 and 30°C.** Black circle and gray bars correspond to data from 8 and 30°C, respectively (data in **Supplementary Table S1**).

FIGURE S2 **Pulse field gel electrophoresis (PFGE) of *L. monocytogenes* strains classified as fast and slow growth at low temperature (8°C)**. Black squares mark strains selected for global gene expression analysis. Details of the strains in **Supplementary Table S1**.

FIGURE S3 **Differentially expressed genes shared between fast and slow groups in response to low temperature grouped by COGs**. The genes were classified into 20 COGs based on their predicted function (COG details in **Figure 2**).

TABLE S1 **Characteristics of the *L. monocytogenes* strains used in the study**. Generation time (Gt) at 8 and 30°C. The lowest 5% and highest 5% of values of Gt were classified as Fast and Slow strains respectively.

TABLE S2 **Microarray data analysis**. Green represents down-regulated genes and red represents up-regulated genes.

TABLE S3 **Primers used in the study**.

Data Sheet S1 **Microarry gpr data**.
