# Supplementary figures and images for: Different Transcriptional Responses from Slow and Fast Growth Rate Strains of Listeria monocytogenes Adapted to Low Temperature
Source: Front Microbiol. 2016 Mar 1;7:229. doi: 10.3389/fmicb.2016.00229 (PMC4772535; doi:10.3389/fmicb.2016.00229)

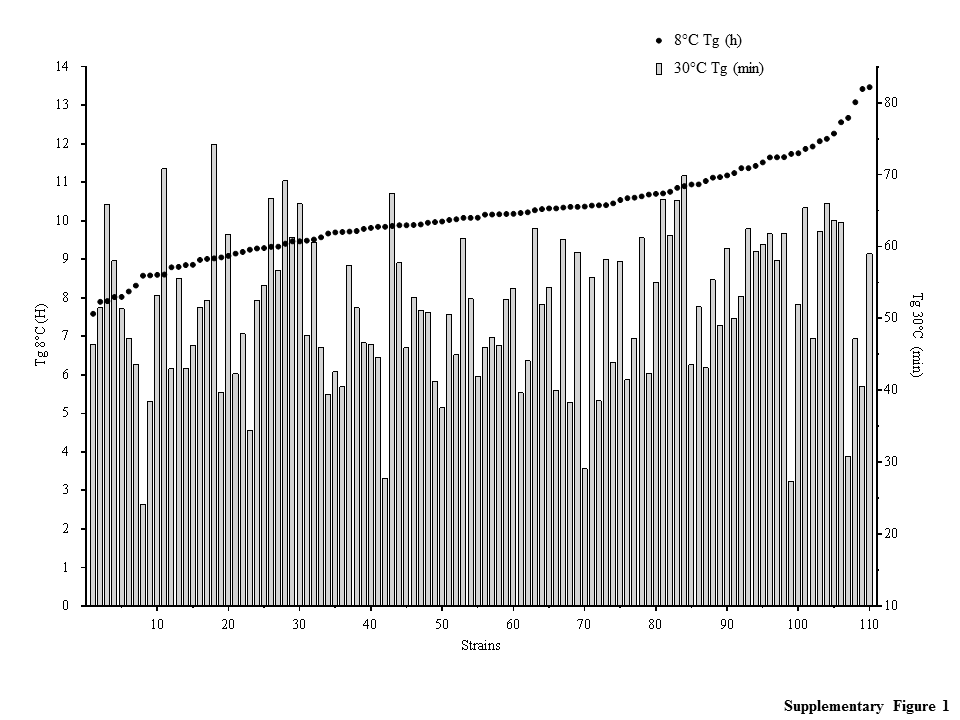

Supplement: FIGURE S1 — Generation time (Gt) distribution at 8 and 30°C. Black circle and gray bars correspond to data from 8 and 30°C, respectively (data in Supplementary Table S1). [file Image_1.TIF]
